# Synchrotron radiation Ca K-edge 2D-XANES spectroscopy for studying the stratigraphic distribution of calcium-based consolidants applied in limestones

**DOI:** 10.1038/s41598-020-71105-8

**Published:** 2020-08-31

**Authors:** Letizia Monico, Laura Cartechini, Francesca Rosi, Wout De Nolf, Marine Cotte, Riccardo Vivani, Celeste Maurich, Costanza Miliani

**Affiliations:** 1 Istituto di Scienze e Tecnologie Chimiche “Giulio Natta” (SCITEC), CNR, Via Elce di Sotto 8, 06123 Perugia, Italy; 2 SMAArt Centre and Department of Chemistry, Biology and Biotechnology, University of Perugia, Via Elce di Sotto 8, 06123 Perugia, Italy; 3 ESRF, Avenue des Martyrs 71, 38000 Grenoble, France; 4 L.A.M.S., CNRS UMR 8220, Sorbonne Université, UPMC Univ Paris 06, Place Jussieu 4, 75005 Paris, France; 5 Department of Pharmaceutical Sciences, University of Perugia, Via del Liceo 1, 06123 Perugia, Italy; 6 Istituto di Scienze del Patrimonio Culturale (ISPC), CNR, Via Cardinale Guglielmo Sanfelice 8, 80134 Napoli, Italy

**Keywords:** Imaging studies, Characterization and analytical techniques, Imaging techniques, Materials science, Analytical chemistry

## Abstract

In Heritage Science, the evaluation of stone consolidation treatments by investigating the nature of in situ newly formed products and their penetration depth within the consolidated matrix is a grand challenge. A number of analytical methods have been proposed, but, currently, most of them are not able to supply a full overview of the spatial, structural and compositional information of the newly formed crystalline and amorphous phases with a submicrometric lateral resolution. Here, we examined, the capabilities of synchrotron radiation (SR)-based two-dimensional X-ray absorption near-edge structure (2D-XANES) spectroscopy at Ca K-edge for determining the structural and compositional properties of the compounds formed after the application of a calcium acetoacetate-based consolidant on a porous carbonatic stone (limestone) and for investigating their stratigraphic distribution at the submicrometric scale length. We evaluated advantages and drawbacks of three Ca K-edge 2D-XANES-based approaches: (i) transmission mode full-field-XANES (FF-XANES) imaging; (ii) micro-X-ray fluorescence (μ-XRF) mapping above the Ca K-edge combined with the acquisition of XRF mode μ-XANES spectra at a limited number of spots; (iii) full-spectral µ-XANES (FS µ-XANES) mapping in XRF mode and its variant called selectively induced X-ray emission spectroscopy (SIXES) mapping. Overall, Ca K-edge 2D-XANES spectroscopy provided accurate qualitative and semi-quantitative information on the newly formed calcium carbonates (i.e., amorphous calcium carbonate, vaterite and calcite) and their stratigraphic distribution at the submicrometric scale, thus opening a new scenario to study the carbonatation process of calcium-based consolidants in limestones.

## Introduction

Stone consolidation is a major challenge for protection of buildings and stone artefacts from weathering and decay, which usually lead to decohesion of the structural elements of the material. Over the last decades, thanks to the progress in material science and nanotechnology, a wide range of novel materials has been developed, with the aim of re-establishing adhesion, cohesion and stability of the damaged stone as well as to improve efficacy and durability of the treatment^1,2^. The effectiveness of a consolidation treatment depends on different aspects, including penetration depth, reaction kinetics and mechanism of formation of the final products, and physical–chemical properties of the consolidant phase in the stone matrix.

In consideration of that, tailored analytical tools and methodologies have been developed to characterize newly synthesized consolidation products and to understand the chemistry behind their performances. Although mechanical and physical tests are based on well-establish methods to evaluate the strengthening action of the consolidation treatment^3^, the structural and compositional characterization of the newly formed phases and the definition of their penetration depth in the stone porosity are more challenging. This is particularly true for products that are based on in situ formation of an inorganic phase with chemical properties similar/equivalent to the stone substrate, thus hampering its investigation. In such context, examples are the new classes of calcium- and silicon-based products, developed to guarantee high chemical and physical compatibility with the stone substrate and designed either to produce inorganic Ca-based phases in carbonate stones or to form new cross-linked silica network in silicate stones^4^. It follows that the in situ characterization of newly formed crystalline and amorphous phases in the stone matrix necessitates analytical spatially-resolved approaches that enable to overcome the limits of conventional (bulk) techniques (such as SEM–EDX, XRD, FT-IR, NMR), which may suffer from low specificity and/or sensitivity, destructiveness and limited lateral resolution^5–7^.

To this purpose, complementary structural and compositional investigation of both crystalline and amorphous phases through the substrate stratigraphy can be achieved by exploiting high lateral resolution micro-spectroscopy techniques. At this regard, micro (µ)-FT-IR (either in reflection or ATR mode) and μ-Raman spectroscopies already demonstrated to be valuable tools to probe the distribution (depth profiles and mapping) of consolidants into carbonate and silicate substrates at lateral resolution down to 5–7 μm^8–10^. Nevertheless, extended overlapping of the vibrational bands in these techniques may hamper clear distinction of the newly formed phases in the microstructure of the stone matrix. In such scenario, synchrotron radiation (SR)-based μ-XRD technique complements the range of the micro-analytical tools available for these applications, providing highly specific identification of the crystalline consolidant phases and their localization at a comparable lateral resolution^11–13^.

More recently, radiation imaging techniques (i.e., X-ray and neutron radiography and tomography) have received great attention as powerful alternative to non-invasively determine the penetration depth and distribution of organic and inorganic consolidants in different types of stone substrates, although no chemical information is provided^14,15^. The coupling of X-ray radiography and tomography with SR sources offers the further advantage of providing measurements at high lateral resolution (down to the submicrometre scale) in a relatively short acquisition time. As an example, SR-based X-ray micro-computed tomography (μ-CT) was used to study the evolution of the 3D microstructure of porosity and connectivity in consolidated limestone at a maximum voxel size of 1 μm^3^ ^16–18^. In addition, neutron radiography and tomography have been successfully used as complementary techniques to X-ray imaging methods to provide high contrast images of the consolidant distribution and penetration depth in the submillimetre range^14,15,19^, while small-angle neutron scattering (SANS) has been exploited for non-invasively probing the open and close porosity distributions in stones at the nanometric scale^20^.

In spite of the great progress achieved in the investigation of consolidation processes of stone substrates by application of the above-mentioned techniques, as a matter of fact they do not permit to obtain simultaneously the chemical identification and the spatial localization at a lateral resolution down to the submicrometre scale. Earlier researches^21,22^ clearly demonstrated that SR-based X-ray absorption near-edge structure (XANES) spectroscopy at Ca K-edge (single point analysis and mapping mode) is a suitable technique for spatially distinguishing among amorphous calcium carbonate (hereinafter referred to as ACC) and different CaCO_3_ polymorphs (i.e., vaterite, aragonite, calcite). Hence, we investigated the use of SR-based Ca K-edge 2D-XANES spectroscopy and µ-XRD mapping for studying the carbonatation process of calcium-based consolidants in limestone substrate at (sub)micrometric lateral resolution.

Specifically, we have tested a newly developed calcium acetoacetate-based consolidant, as a precursor of CaCO_3_^23^. Earlier studies have shown that different metastable calcium carbonate phases (i.e., first ACC followed by vaterite) can precede the formation of stable calcite in calcium-based consolidants (including those based on calcium acetoacetate) and that the formation of calcite is favored with increasing relative humidity (RH)^24–27^. Thus, for these specific systems, it is of major interest providing detailed information on the nature and distribution of the newly formed calcium carbonate crystalline phases and ACC directly in the stone porosity and on the environmental conditions at which they preferentially form.

To this aim, we evaluated three different Ca K-edge 2D-XANES-based approaches: (i) transmission mode full-field (FF)-XANES imaging; (ii) μ-XRF mapping just above the Ca K-edge combined with the collection of XRF mode μ-XANES spectra at a limited number of locations; (iii) full-spectral (FS) µ-XANES mapping in XRF mode. The FS µ-XANES mapping data were also used to assess the applicability of the so-called selectively induced X-ray emission spectroscopy (SIXES) method, which consists in recording μ-XRF maps at a few energies around the absorption edge of a specific element, in our case the Ca K-edge^28^.

The study was carried out on two cross-sections obtained from fragments of a calcium acetoacetate-based consolidated limestone substrate (Lecce stone). Strengths and limits of each approach are here presented and discussed in terms of analytical performance, determined as the best compromise between data quality, acquisition time and radiation damage, as well as preparation of the analyzed samples.

## Materials and methods

### Calcium acetoacetate-based consolidant product

We have selected a calcium acetoacetate-based consolidant developed within the FP7 funded project HEROMAT^23,29^. With the aim to obtain different CaCO_3_ phases to be used as reference compounds, the consolidant was left to react and aged at controlled conditions of temperature and RH. About 10 g of the water-based consolidant formulation with 9.6 wt.% of calcium acetoacetate (hereinafter designated CFW) was poured into a Petri dish. After complete evaporation of the solvent (i.e., after about 1 week), it was moved into a sealed vessel at RH = 50%, then stored in an oven (T = 40 °C) for 5 months. The RH level was achieved by filling the lower section of the vessel with a saturated aqueous solution of Mg(NO_3_)_2_·6H_2_O (Sigma Aldrich).

### Lecce stone mock-up

Lecce stone was chosen as calcareous substrate with high porosity (ranging from 31% to 45%)^30^. Lecce matrix is mainly composed of calcite and dolomite [CaMg(CO_3_)_2_] with accessory minerals, such as quartz (SiO_2_) and phengite (KAl_2_[AlSi_3_O_10_](OH)_2_ K(Mg, Fe^2+^)(Al, Fe^3+^)[Si_4_O_10_](OH)_2_), all identified by SR µ-XRD mapping (Supplementary Information, Fig. S1). A mock-up (3 × 2 × 6 mm^3^) was treated by immersion in CFW (formulation with 31.75 wt.% of calcium acetoacetate) with 0.05 wt.% ethylenediamine (employed as a catalyst). After 24 h, the sample was taken out of the consolidant solution, moved in a Petri dish and stored in a sealed vessel at T = 40° C and RH = 50% for 5 months. Such conditions were achieved using the same procedure as previously described for the calcium acetoacetate-based consolidant product (see “Calcium acetoacetate-based consolidant product”).

### FT-IR spectroscopy

Transmission mid-FT-IR spectrum of the CFW consolidant powder was recorded by means of a Jasco FT/IR-470 plus spectrophotometer, comprising a Globar IR radiation source, a Michelson interferometer, and a pyroelectric deuterated triglycine sulfate (DTGS) detector. The FT-IR spectrum was collected from a pellet of KBr (Sigma Aldrich) in the range 400–4,000 cm^-1^ and with a spectral resolution of 2 cm^-1^.

### XRD

XRD measurements of the CFW consolidant powder were performed by a PANalytical X'PERT PRO diffractometer using the CuK_α_ radiation in Bragg–Brentano reflection mode. The goniometer was equipped with an X'Celerator fast detector. Diffraction patterns were collected in the 17°–100° 2θ range, using a 0.017° angular step, and 100 s counting time per step. A Rietveld refinement procedure was performed in order to estimate the weight fraction of the amorphous phase and to give an average evaluation of microstructure of the sample, in terms of mean size of crystallite domains (volume weighted) and residual lattice strain, as a measure of lattice defects.

For the estimation of the weight fraction of the amorphous phase, a weighted amount of crystalline silicon powder (12.5% w/w) was added to the sample as internal standard.

The Rietveld refinement procedure involved the refinement of scale factors, background, unit cell, and profile parameters. The refinement method can determine the weight fractions of crystalline phases by the values of refined scale factors. The presence of an amorphous phase produces an overestimation of these weight fractions, that can be rescaled by the knowledge of the added weight fraction of crystalline internal standard according to the following equation:

1 $${\text{W}}_{{\text{i,c}}} = {\text{W}}_{{\text{i}}} \frac{{{\text{W}}_{{\text{s,w}}} }}{{{\text{W}}_{{\text{s}}} }}\left( {\frac{1}{{1 - {\text{W}}_{{\text{s,w}}} }}} \right)$$

where W_i,c_ is the rescaled, correctly estimated, weight fraction of phase i, W_i_ and W_s_ are the weight fractions of phase i and the internal standard, respectively, obtained by the refinement procedure, and W_s,w_ is the experimental weight fraction of the internal standard^31^.

The weight fraction W_a_, associated to the amorphous phase, is then calculated by the following equation:

2 $${\text{W}}_{{\text{a}}} = 1 - \sum\nolimits_{{\text{i}}} {{\text{W}}_{{\text{i,c}}} }$$

By this procedure an accuracy of a few percent is usually achieved^32^.

Rietveld refinements were performed by means of the software GSAS^33^. At the end of refinements, all calculated shifts were less than their standard deviations. Agreement factors for the last refinement cycle were R_w_ (weighted residual) = 0.082 and R_p_ (profile residual) = 0.060.

The mean size of crystallite domains (volume weighted) and residual lattice microstrain were assumed to contribute isotropically to the XRD line broadening, and were estimated by refining the X and Y parameters of the modified pseudo-Voigt function implemented in the GSAS package^34^, by following the procedure earlier described^35^. The instrumental contribution to the peak broadening was previously evaluated by the Rietveld refinement of the profile of lanthanum hexaboride, as an external standard. The coherent domain sizes (*Dv*), and microstrain values (ε) were estimated using the following equations^33^:

3 $$Dv = { 18}00\;\lambda/\pi {\text{X}}$$

4 $$\varepsilon = \, (\pi/{18}000)\left( {{\text{Y}} - {\text{Y}}_{{\text{i}}} } \right)$$

where Y_i_ is the instrumental contribution, obtained by the refinement of lanthanum hexaboride pattern. The crystal structure of vaterite was taken from ref.^36^.

### SR-based µ-XRF mapping and XANES spectroscopy at Ca K-edge

Calcium speciation measurements of the powder of the consolidant products and of the consolidated Lecce stone mock-up were performed at the FF-XANES and scanning X-ray micro-spectroscopy (SXM) end stations hosted at beamline ID21 of the European Synchrotron Radiation Facility (ESRF, Grenoble, France)^37^.

The powders were spread as a thin layer (~ 10–20 µm in thickness) on S-free adhesive tape and covered with a foil of ultralene. For the consolidated Lecce stone mock-up, measurements were carried out on two cross-sections prepared from fragments taken from the mock-up itself (sizes of ~ 3 × 2 mm^2^), embedded into polyester resin and then polished down to a thickness of either 80 µm (sample L_1_) or 150 µm (sample L_3_). The X-ray energy was tuned by employing a fixed exit double-crystal Si(111) monochromator at both the FF-XANES and SXM end stations. The energy calibration was performed using calcite as standard and by setting the position of the peak maximum of its first-order derivative spectrum at 4,046.06 eV. The observed peak energy shifts in the XANES spectra with respect to those earlier reported reflect differences in monochromator calibrations^22,38^.

Ca K-edge FF-XANES imaging in transmission mode was carried out using an unfocused beam (size of ~ 1.5 × 1.5 mm^2^, defined using slits). A Lu_2_SiO_5_:Tb scintillator (located at a distance less than 2 mm, downstream the sample) was used to convert X-ray transmission images into visible images. A 10 × optical objective was employed to magnify the image onto a CMOS camera (PCO edge, Germany) with a pixel size of ~ 0.65 × 0.65 μm^2^ and giving a lateral resolution of ~ 1.4 μm. The maximum field of view (FOV) was around 1,000 × 1,000 μm^2^. A stack of 549 X-ray radiographs was recorded, while tuning the X-ray energy across the Ca K-edge with the following energy step sizes: (i) 5 eV in the 3,938–4,018 eV and 4,238–4,398 eV range, (ii) 0.3 eV in the 4,018–4,138 eV region and (iii) 1 eV in the 4,138–4,238 eV range. For each energy, images were acquired with and without the sample (for flat-field correction), which requires moving the sample in and out at every energy. A total dwell-time of about 37 s per energy was required, which includes motion time, acquisition time of the images of both the reference (~ 0.9 s) and the sample (~ 29 s), overhead time and time to save the recorded data. Further details of the experimental setup and full-field XANES measurements at ID21 can be found elsewhere^39^.

An in-house developed Python script was employed for image alignment of the XANES stacks, while the TXM-Wizard software package^40^ was used to produce phase maps of different calcium-based compounds and CaCO_3_ polymorphs. After determination of the edge-jump, noise filtering and normalization (performed using two linear functions, one in the pre-edge and one in the post-edge region), semi-quantitative Ca phase maps were obtained by describing the XANES spectra at each pixel as least squares linear combination (LSLC) fit of a set of XANES spectra of seven calcium reference compounds with the following constraints: upper bounds of 1 and lower bounds of 0 for each component and the sum of all components forced to be equal to 1. The combination yielding the best fit quality (checked taking into account the chi-square, reduced chi-square and R-factor values) was chosen as the most likely set of Ca-compounds present at that location (see Supplementary Information, Figs. S2-S3 and Table S1 for details).

At the SXM end station, macro-XANES spectra were collected using a 200 µm beam, while µ-XANES and µ-XRF map acquisitions were performed by focusing the beam with a Kirkpatrick-Baez mirror system down to a probe of 0.7 × 0.3 μm^2^ (h × v). XRF signals were collected at an angle of 69° with respect to the incident beam direction by means of a silicon drift detector (XFlash 5100, Bruker). The beam intensity was recorded for each pixel thanks to a photodiode upstream the sample. μ-XRF mapping experiments were performed by employing a monochromatic primary beam of fixed energy around the Ca K-edge while raster scanning the sample. The PyMca software^41^ was used to fit the XRF spectra and to separate the contribution of different elements. The same software along with the available online software developed by The Center for X-Ray Optics^42,43^ were employed to calculate the transmission % value and the X-ray attenuation length at the Ca K-edge energy of the cross-sections obtained from the consolidated Lecce stone mock-up.

From the μ-XRF maps, points of interest were selected for the acquisition of macro- and µ-XANES spectra, that were acquired in XRF mode by scanning the primary energy around the Ca K-edge (total point/energy step numbers: 549; exposure time: 0.1 s per point; 3 scans) with the same energy step sizes used for recording the FF-XANES stack (see above for details). The normalization and the LSLC fit of each single point spectrum against a library of XANES spectra of Ca-reference compounds was performed by means of the ATHENA software^44^.

FS μ–XANES mapping investigation of a region of interest of sample L_1_ was performed by recording a stack of 74 µ-XRF maps (exposure time: 40 ms/pixel), while tuning the X-ray energy across the Ca K-edge with the following steps: (i) 4 eV in the 4,012–4,040 eV and 4,080–4,120 eV range, (ii) 0.5 eV in the 4,040–4,046 eV region, (iii) 1 eV in the 4,046-4074 eV range, (iv) 2 eV in the 4,074–4,080 eV range and (v) 6 eV in the 4,120–4,198 eV region. Single pixel XANES spectra were normalized with PyMca and fitted by LSLC, following the same procedure and constraints as for FF-XANES.

To illustrate what the SIXES approach could have offered, a stack of 8-energy µ-XRF maps was extracted from the FS μ–XANES dataset. The highest energy map at 4,198.0 eV was used for normalizing the other seven maps, whose energy values were chosen from the XANES spectra of the corresponding seven reference compounds (see Supplementary Material, Figs.S2 and S4, for further details). Each single pixel of the normalized SIXES stack was fitted as a LSLC of XANES spectra of seven Ca-based reference compounds (reduced to the same 8 energies). The same LSLC boundary constraints as for FS μ–XANES mapping and FF-XANES imaging were applied.

During the experiment, we performed preliminary XANES measurements at varying fluences on powders of CaCO_3_ polymorphs and a fragment obtained from the not treated Lecce stone mock-up to ensure that, under the employed conditions, spectral profiles were not altered by damages due to X-ray beam exposure.

## Results

### Structural and compositional characterization of the calcium acetoacetate-based consolidant product

**Figure 1:**
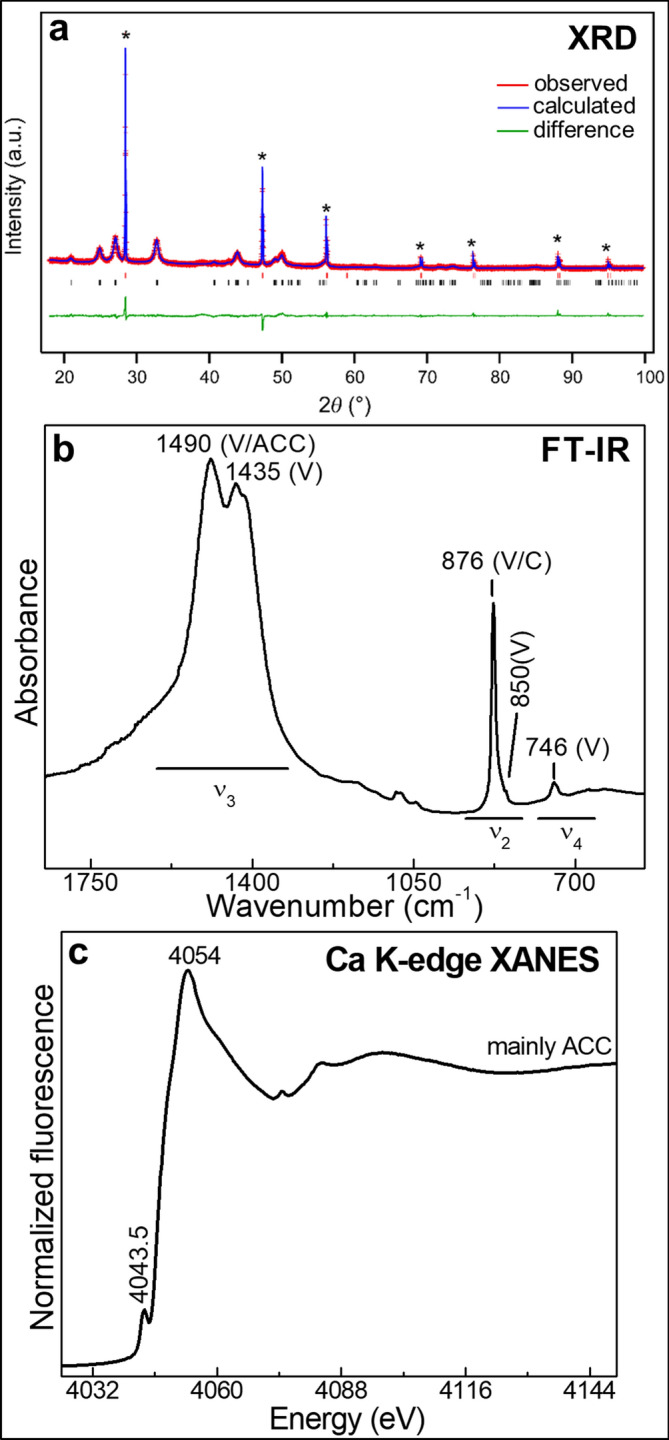
Bulk analysis of CFW consolidant after storing at RH = 50% (T = 40 °C, 5 months): **(a)** Rietveld refinement performed by GSAS^33^, **(b)** FT-IR and **(c)** Ca K-edge macro-XANES spectrum (unfocused beam, 200 µm pinhole) normalized by ATHENA^44^. In **(a)**, the peaks marked with an asterisk are related to the silicon added as internal standard. Red and black vertical bars at the bottom indicate the calculated positions of peaks for silicon and CFW consolidant, respectively. In **(b)**, letters denote: *V* = vaterite, *ACC = * amorphous calcium carbonate, *C* = calcite.

We started by performing a bulk characterization of the CFW powder by XRD and FT-IR spectroscopy complemented with single point XRF mode Ca K-edge macro-XANES measurements (Fig. 1). The Rietveld refinement of the XRD profile (Fig. 1a) points to the presence of a nanosized vaterite phase, characterized by an average crystallite size of 18 nm and a moderate microstrain (0.3%). No other CaCO_3_ polymorphs, such as calcite and/or aragonite, were detected. In addition, the presence of 62% w/w of amorphous phase was estimated by XRD.

Calcite, vaterite, aragonite and ACC present similar FT-IR spectra, but with subtle differences in band positions, widths and relative intensities, which can be used to differentiate them^27,45,46^. In Fig. 1b, the clear presence of vaterite is pointed out by the intense band at 1,435 cm^-1^ [asymmetric stretching (ν_3_) of CO_3_^2-^], the shoulder peak at 850 cm^-1^ [out-of plane bending vibration (ν_2_) of CO_3_^2-^], and the signal at 746 cm^-1^ [in-plane bending mode (ν_4_) of CO_3_^2-^]^27,45,46^. The band at about 1,490 cm^-1^ [ν_3_(CO_3_^2-^)] can be attributed both to ACC and vaterite, while that at 876 cm^-1^ [ν_2_(CO_3_^2-^)] is assignable both to calcite and vaterite^27,45,46^. The absence of the peaks at 1709 cm^-1^, 1,580 cm^-1^ and the CH stretching vibrational modes in the 3,000–2,800 cm^-1^ range (not shown) indicates that the consolidant precursor calcium acetoacetate is not present anymore in CFW. Based on such data, the amount of amorphous material estimated from XRD can be reasonably assigned to ACC.

**Figure 2:**
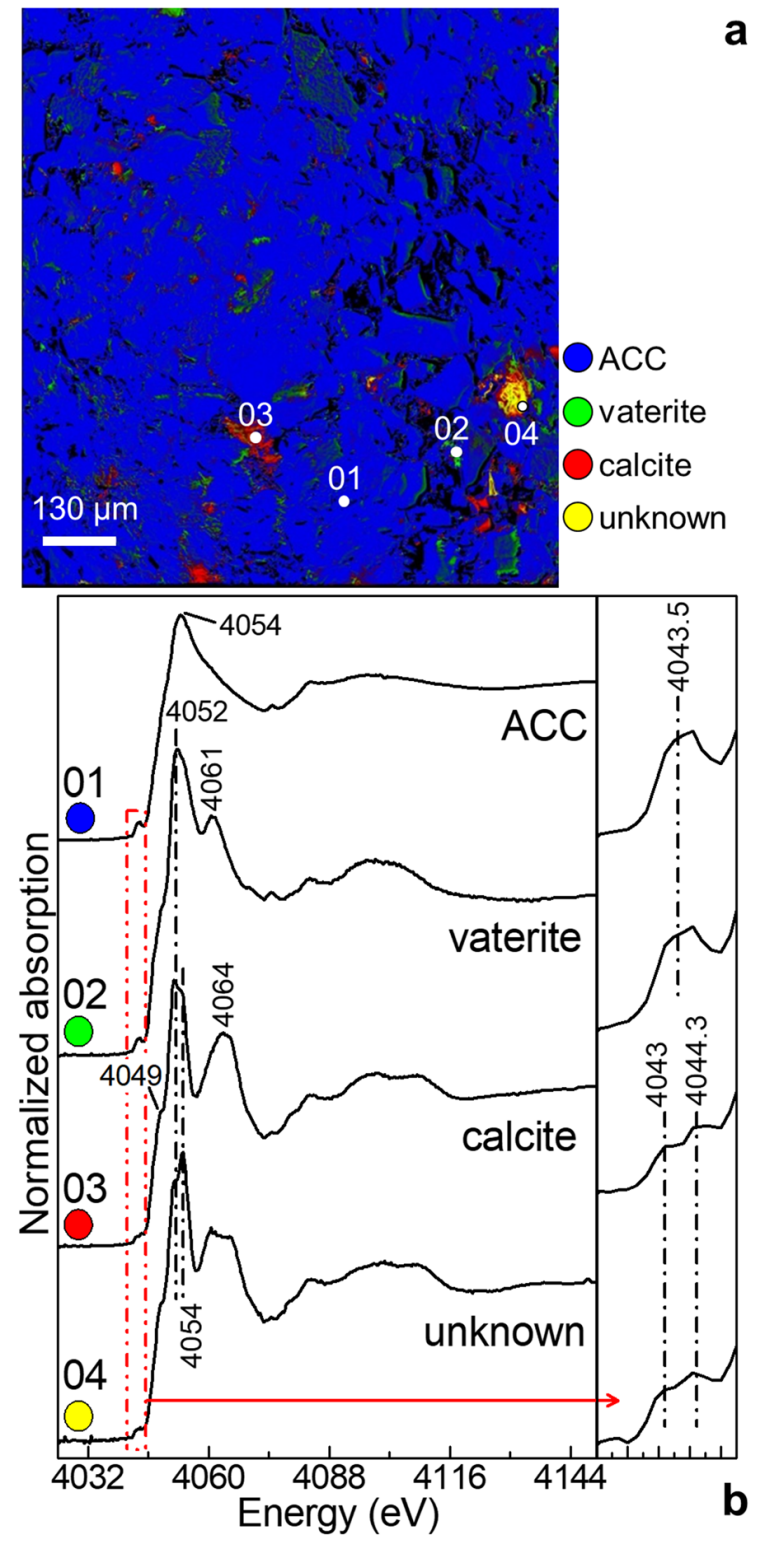
**(a)** Composite Ca phase maps obtained from the CFW consolidant aged at RH = 50% (∼10–20 µm in thickness) by least squares linear combination (LSLC) fit of the Ca K-edge FF-XANES stack, using the spectra of (red) calcite, (green) vaterite, (blue) ACC and (yellow) an unknown Ca-based compound as references [map size (h × v): 975.65 × 977.6 µm^2^]. **(b)** Ca K-edge XANES spectra (black) extracted from the areas shown by white circles in **(a)**, and where the relative amount percentage of each phase is ≈ 95–100% [average pixel number: 5–20; pixel sizes (h × v): ~ 0.65 × 0.65 μm^2^]. The inset on the right-side shows the pre-edge absorption region pointed out by the red dotted rectangle. Data were processed by means of TXM-Wizard^40^.

The single point XRF mode Ca K-edge XANES spectrum obtained using an unfocused beam (200 µm pinhole) (Fig. 1c), resembles that of ACC, as shown by the presence of a single pre-edge peak at 4,043.5 eV (1s → 3d transition) along with a single large post-edge band at about 4,054 eV (see Fig. 2 and Supplementary Information, Fig. S2, for a comparison with the spectra of vaterite and calcite)^22^. It is worthwhile to mention that the Ca K-edge XANES spectra of ACC and of a number of organo calcium compounds are very similar^21,47,48^, thus making an unequivocal discrimination between calcium acetoacetate and ACC challenging. Nevertheless, based on the XRD and FT-IR analysis (Fig. 1a,b), it is reasonable to assume that only ACC is present. Overall, the complementarity among bulk XRD, FT-IR and single point XRF mode Ca K-edge macro-XANES spectroscopy permitted us to highlight that two phases (i.e., vaterite and ACC) are the main constituents of the CFW powder.

In order to better understand the compositional heterogeneity of the CWF powder, we have further investigated the sample at the submicrometre scale by performing Ca K-edge FF-XANES imaging in transmission mode. Figure 2a shows that the powder is predominately composed of ACC (blue) but with some distinct areas where crystals of variable shape and size (below ∼80–90 µm) of calcite (red), vaterite (green) and an additional Ca-based phase of uncertain attribution (yellow) have formed. From the spectral point of view (Fig. 2b), calcite is clearly identified through two weak pre-edge peaks at 4,043 and 4,044.3 eV (1 s → 3d transition), the shoulder at 4,049 eV (1s → 4s transition) and the edge/post-edge signals at 4,052 and 4,064 eV, while vaterite features a single broad pre-edge peak at 4,043.5 eV (1s → 3d transition) along with two post-edge bands at 4,052 and 4,061 eV^21,22^. The Ca-based phase of uncertain attribution is characterized by a XANES spectrum very similar to that of calcite but with small differences in the relative intensity and shape of the post-absorption bands positioned in the 4,050–4,070 eV region. Based on earlier studies, these changes might be attributable either to the presence of calcite crystals having a different orientation with respect to the polarized nature of the X-ray beam^21,49^ or to an initially formed CaCO_3_ phase with a short-range order similar to calcite, in which the carbonate coordination sphere is at first distorted but then adopts the octahedral symmetry typical of calcite^38^.

### Calcium-speciation investigations of the CFW consolidated Lecce stone mock-up

The capabilities of FF-XANES imaging to discriminate among different CaCO_3_ polymorphs and to probe their distribution at the submicrometre scale have then been exploited to investigate a Lecce stone mock-up treated with the CFW consolidant. In order to determine the suitable sample thickness for performing transmission mode measurements, we investigated two thin sections having thickness of 80 µm (sample L_1_) and 150 µm (sample L_3_).

**Figure 3:**
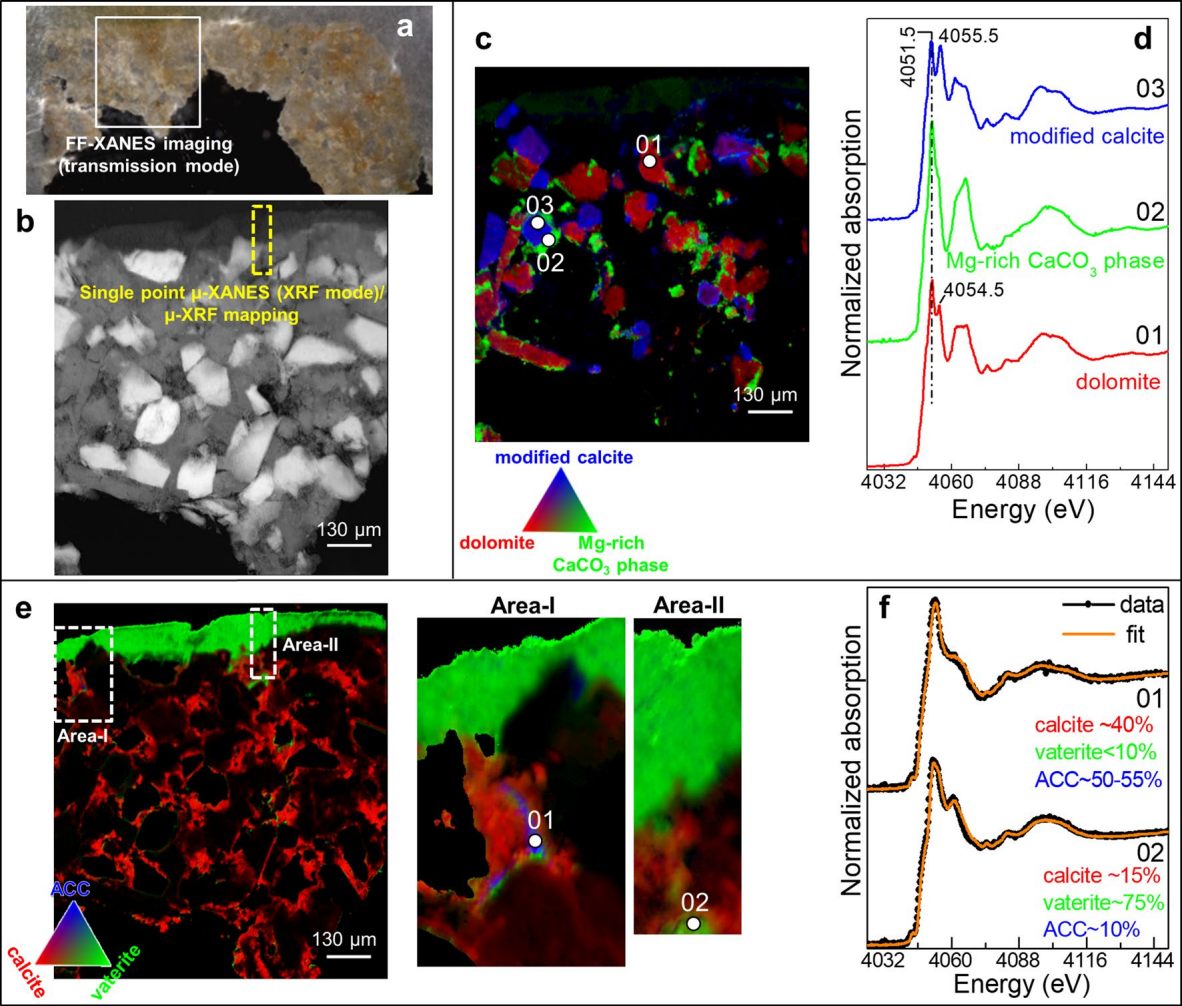
**(a)** Microphotograph of the cross-section L_1_ (~ 80 µm in thickness) obtained from the Lecce stone mock-up consolidated with CFW (RH = 50%, T = 40 °C, 5 months). **(b)** Transmission image recorded at 3,918 eV from the area shown in **(a)**. RGB Ca phase maps of **(c)** dolomite/Mg-rich CaCO_3_ phase/modified calcite and **(e)** calcite/vaterite/ACC [FOV: ~ 1,000 × 1,000 µm^2^; pixel sizes (h × v): ~ 0.65 × 0.65 µm^2^]. **(d,f)** Ca K-edge XANES spectra (black) extracted from regions of interest of the FF-XANES stack shown in **(c,e)** (average pixel number: 5–20) and corresponding LSLC fit result of different Ca-based compounds (orange). Data were processed by employing TXM-Wizard^40^.

The analysis, performed in a region of interest of the 80 µm thinned cross-section (Fig. 3a,b), confirms that the Lecce matrix is mainly composed of calcite (Fig. 3e, red) with crystals of variable shape and size of dolomite (Fig. 3c, red), a Mg-rich CaCO_3_ phase (green), and one additional Ca-based compound of uncertain attribution (blue). The XANES spectrum of the unknown phase (called modified calcite; Fig. 3d, blue line) is characterized by a white line positioned at the same energy (4,051.5 eV) as for that of dolomite (red line) and the Mg-rich CaCO_3_ phase (green line). However, it shows slight differences in the shape, relative intensity and position of the post-edge bands located in the 4,059–4,071 eV range and at 4,055.5 eV. These changes are possibly related to structural changes of CaCO_3_-based crystals, due to the substitution with different Mg abundance and/or to the neighboring presence of Si-containing compounds, such as quartz and phengite (Supplementary Information, Fig. S1)^50–52^.

XANES measurements performed on the untreated Lecce stone mock-up (results not shown) did not reveal the presence of either vaterite or ACC, meaning that the penetration depth and chemical evolution of the CFW consolidant in the CaCO_3_-based substrate can be evaluated by monitoring the distribution of these phases.

Based on the results shown in Fig. 3e, vaterite (green), formed by the consolidant application, is present as a uniform layer of about 60–100 µm in thickness at the surface of sample L_1_ (Area-I, Area-II). In few porosities of the calcite-based matrix (red), both ACC (Area-I, blue) and vaterite have been found. In these regions, the relative abundance of ACC and vaterite (Fig. 3f and Supplementary Information, Table S1) achieved relative amounts up to around 50–55% (pt. 01) and ~ 75% (pt. 02), respectively. Complementary SR µ-XRD mapping investigations (Supplementary Information, Fig. S1) confirmed that the distribution of vaterite and calcite is similar to that obtained by FF-XANES imaging. Nevertheless, such analysis did not provide any meaningful information about the presence of amorphous phases.

**Figure 4:**
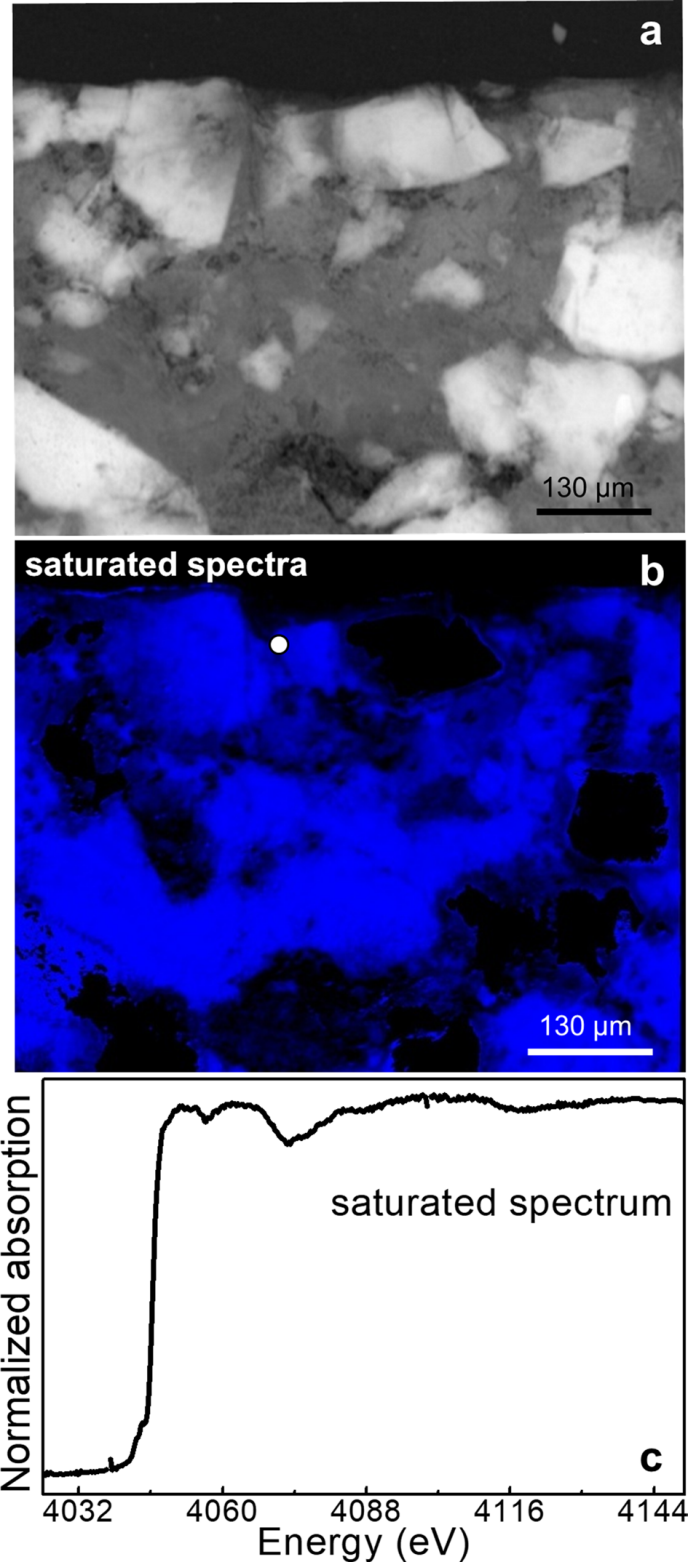
**(a)** Transmission image recorded at 3,918 eV from the cross-section L_3_ (~ 150 µm in thickness) obtained from the Lecce stone mock-up consolidated with CFW (RH = 50%, T = 40 °C, 5 months). **(b)** Saturated Ca phase map obtained by FF-XANES imaging and **(c)** corresponding Ca K-edge XANES spectrum [average pixel number: 5; pixel sizes (h × v): ~ 0.65 × 0.65 μm^2^] extracted from the area indicated by the white circle in **(b)**. Data were processed using TXM-Wizard^40^.

Overall, our results show that Ca K-edge FF-XANES imaging in transmission mode can be successfully employed for studying heterogeneous limestone samples, permitting to distinguish various crystalline and amorphous CaCO_3_-based compounds with high specificity, high lateral resolution (~ 1.4 µm) and large FOV (orders of mm^2^). However, it is known that the success of this kind of investigations strongly depends on the thickness and composition of the analyzed sample^53–55^. As shown in Fig. 4, the spectra extracted from the FF-XANES imaging dataset obtained from the thicker cross-section L_3_ (~ 150 µm in thickness) shows strong distortions, thus making not possible to obtain any meaningful information about the identification and spatial distribution of different Ca-based phases from the analysis. This issue results from the low transmission intensity percentage of the X-ray beam at the Ca K-edge (4,038 eV). Actually, considering an average density = 2.7 g/cm^3^ for the Lecce stone mock-up (mainly composed of CaCO_3_), the maximum transmission % value calculated at the Ca K-edge is around 2% for sample L_3_. With the present sample composition, an ideal thickness of ≤ 30 µm would have been required to obtain a transmission % value above 40%. Here, the thinner cross-section that could be obtained, L_1_ (~ 80 µm in thickness), gave a maximum transmission % value of about 15%. This was not optimal, but allowed obtaining reliable FF-XANES results.

**Figure 5:**
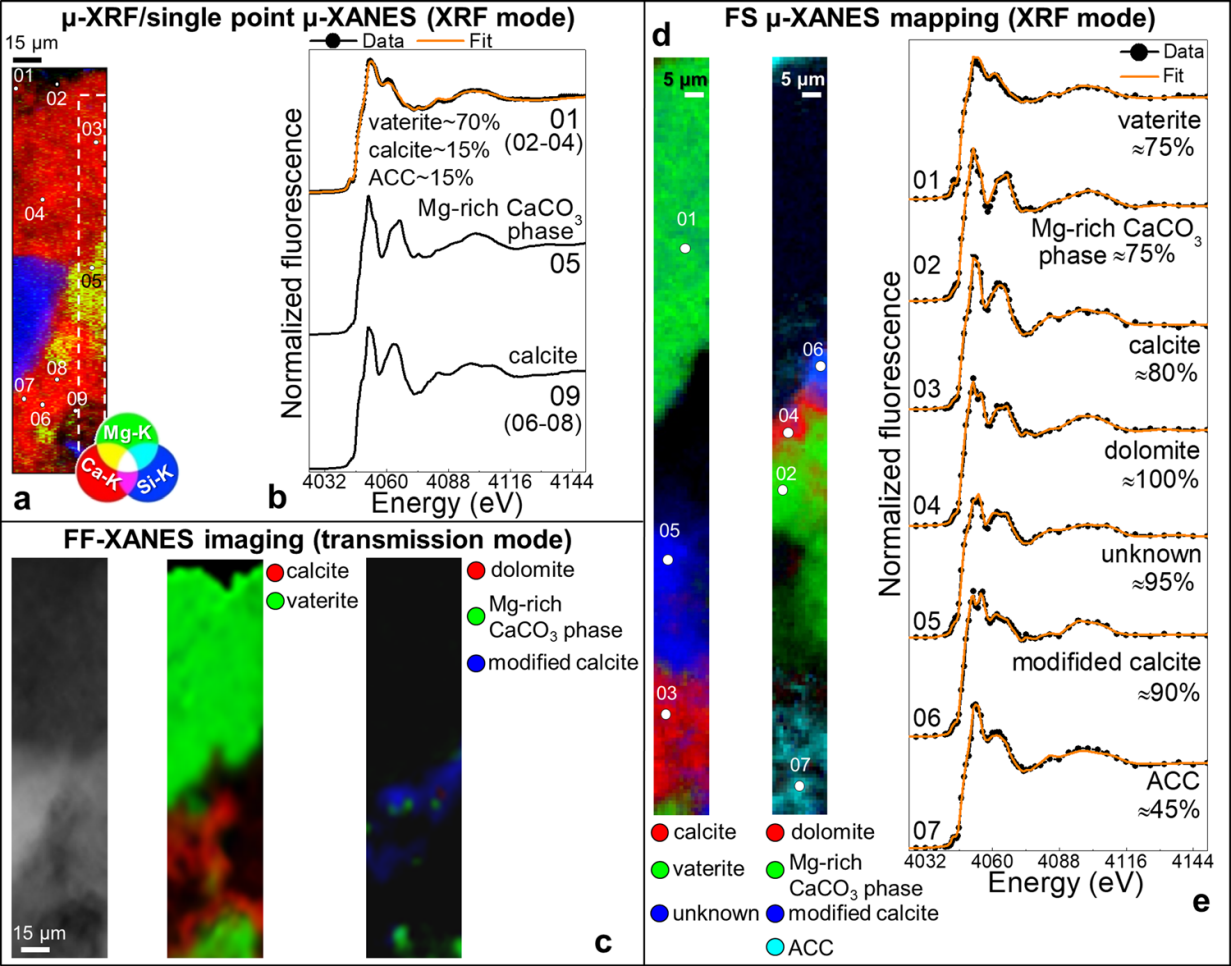
**(a)** RGB µ-XRF maps of Ca/Mg/Si recorded from a region of interest of sample L_1_ [map size (h × v): 48 × 206 µm^2^; step size (h × v): 1.5 × 0.6 µm^2^; exp. time: 50 ms/pixel; energy: 4,400 eV] and **(b)** Ca K-edge µ-XANES spectra recorded from the spots shown in **(a)** (see Fig. 3b for the location of the mapped area). **(c)** Magnification of a region of sample L_1_ analyzed by FF-XANES imaging: (from left) transmission image recorded at 3,918 eV and corresponding RGB Ca phase maps of calcite/vaterite and dolomite/Mg-rich CaCO_3_ phase (cf. Fig. 3e to see the entire analyzed area). **(d)** Composite FS µ-XANES maps of (left) calcite/vaterite/unknown Ca-based compound and (right) dolomite/Mg-rich CaCO_3_-phase/modified calcite/ACC recorded from the area indicated by the white-dotted rectangle in **(a)** [map size (h × v): 15 × 205 µm^2^; step size (h × v): 1 × 1 µm^2^; exp. time: 40 ms/pixel]. **(e)** Ca K-edge µ-XANES spectra (black) extracted from region of interests of the FS μ-XANES stack shown in **(d)** (average pixel number: 3–5) and corresponding LSLC fit result of different Ca-based compounds (orange) (only the relative amount percentage of the main phase is reported; for details see Supplementary Information, Table S1). Data were processed by means of PyMca^41^, ATHENA^44^ and TXM-Wizard^40^.

Problems typically encountered in transmission mode spectroscopy for the analysis of the stratigraphy of thick, non-transparent samples, can be overcome by performing XRF mode XANES analysis^55^. For both the thinner (L_1_) and thicker (L_3_) cross-sections of the consolidated Lecce stone mock-up, we have analyzed a region of interest at the SXM end station, by acquiring a µ-XRF map above the Ca K-edge (i.e., 4,400 keV). Points of interest were selected from this map (in particular from the elemental distribution of Ca, Mg and Si) and Ca K-edge µ-XANES spectra were acquired in XRF mode at these locations (Fig. 5a). Since similar results have been obtained for the two analyzed samples, only those related to L_1_ are shown in Fig. 5. µ-XANES spectra (Fig. 5b) clearly discriminate among the presence of different CaCO_3_-based compounds. In line with the FF-XANES imaging results (Fig. 5c), the data show that the matrix is mainly composed of calcite (Fig. 5b, pts. 06–09), while vaterite is present in abundance up to 70% in the uppermost side of the cross-section (pts. 01–04). The spectrum collected from a Mg-rich region (pt. 05) highlights the presence of a Mg-rich CaCO_3_ phase.

**Figure 6:**
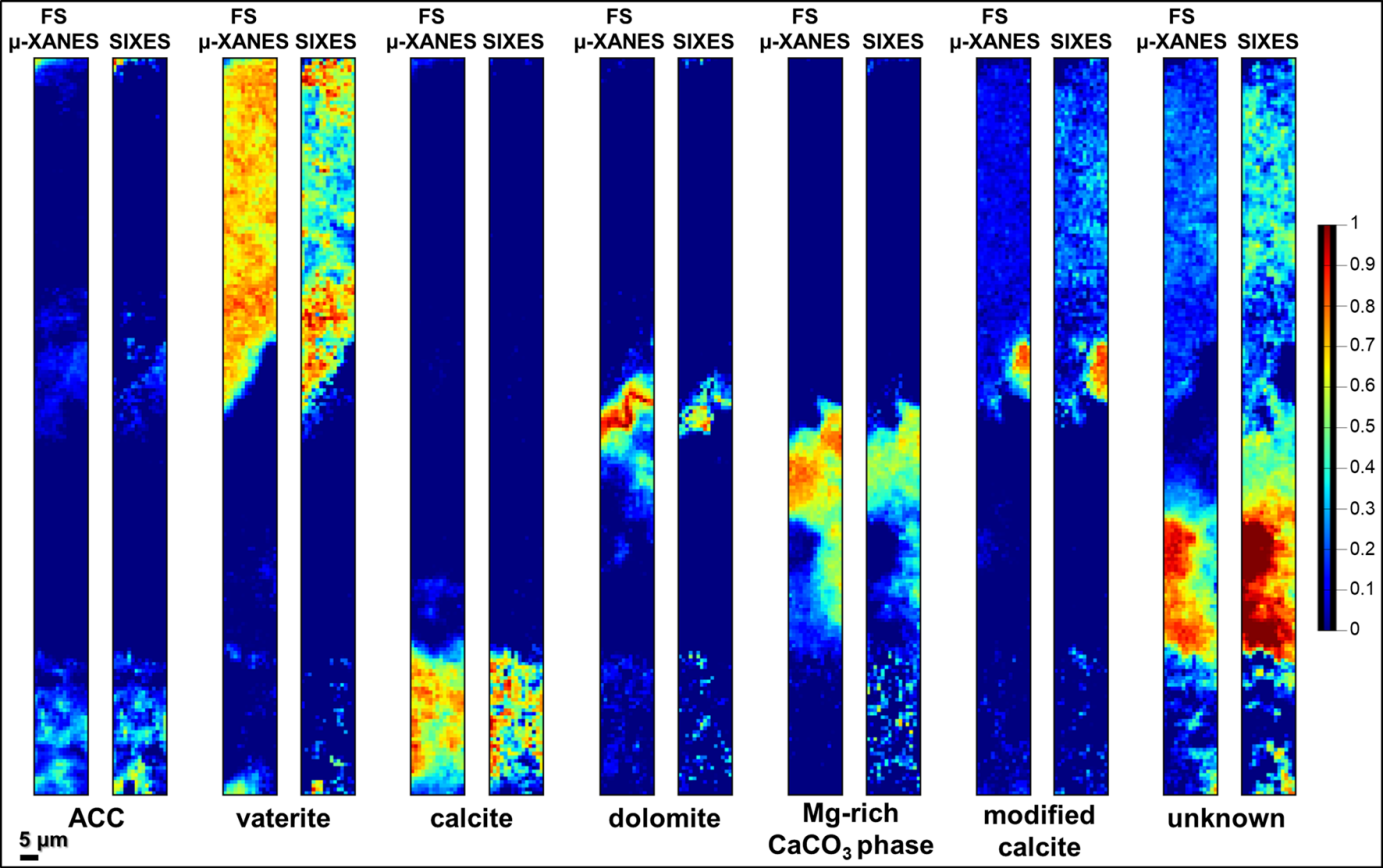
Comparison between the normalized distribution maps of different Ca-based phases obtained by LSLC fit of: (left) FS μ-XANES stack (n. 74 energies) (cf. Fig. 5d,e) and (right) SIXES stack (n. 8 energies) (see Supplementary Information, Figs. S2 and S4, for further details).

Alternatively to FF-XANES imaging, 2D-XANES mapping was also carried out in XRF mode (FS µ-XANES). This method consists in acquiring the same µ-XRF map, excited at tens of energies, across the Ca K-edge. The region of interest of the sample L_1_ selected for FS µ-XANES mapping is shown in Fig. 5a and results are presented in Fig. 5d,e and Fig. 6 (leftmost panels). The distribution of different Ca-based phases is in line with the one resulting from the XRF mode single point µ-XANES analysis and the FF-XANES imaging experiment (Fig. 5b,c), with mainly vaterite (up to ~ 75%) and minor relative abundances of ACC (~ 10–45%) present in the uppermost 50–60 µm of the sample and in the porosity of the calcite-based matrix (up to ~ 80%) (see Supplementary Information, Table S1, for further details). The higher representativeness of the FS µ-XANES mapping data compared to the XRF mode single point µ-XANES ones, allowed us to reveal the presence of other Ca-based phases, some of them already identified by FF-XANES imaging (cf. Figs. 2, 3 and 5c), including dolomite and the modified calcite-phase (Fig. 5d,e). The analyses reveal also the presence of one additional unknown Ca-compound, characterized by XANES spectral features similar to those observed for the CFW powder (cf. Figs. 2 and 5e, unknown). In addition, a Mg-rich CaCO_3_ region is visible only in the FS µ-XANES map but not in the FF-XANES images (Fig. 5c,d, rightmost panels). This might be due to the different geometry of SXM and FF-XANES experimental setups as well as to the heterogeneity in the thickness of the sample investigated (see “Discussion” for further details).

Finally, in order to assess the feasibility of a faster SIXES mapping approach, a reduced stack of eight energy maps was extracted from the non-normalized FS µ-XANES dataset of sample L_1_ and fitted at each pixel as a LSLC of the seven reference Ca-based compounds. The corresponding Ca phase maps are shown in Fig. 6. At the cost of more noise, the distribution of the seven species matches very well with those obtained using the 74-energy FS µ-XANES dataset. The main regions are well identified, however with slight differences in terms of species concentration. Fig. S4 (Supplementary Information) further quantifies the differences between the SIXES and FS µ-XANES mapping results. In brief, for ACC, calcite, dolomite, modified calcite and Mg-rich CaCO_3_ phase, the results obtained via the two approaches are in good agreement with more than 75% of the pixels showing a difference in terms of fitted concentration of each species less than ± 0.15. For the unknown phase and vaterite, differences are more pronounced. In the area where vaterite is the main component (upper layer), its concentration, as determined by SIXES mapping, is underestimated, while that of the unknown species is overestimated.

## Discussion

In what follows, the results described in “Results” will be used to discuss and compare the technical features of the FF-XANES imaging, FS µ-XANES mapping and SIXES mapping approaches in terms of acquisition times, spectral and lateral resolution, data representativeness, effects related to their detection geometry and thickness of the analyzed samples.

As reported in Table 1, by using the FF-XANES setup (Figs. 3 and 4), the acquisition of 549 incident energy-stack of images around the Ca K-edge (3,938–4,398 eV) from large areas (~ 1,000 × 1,000 µm^2^) of samples L_1_ and L_3_ with micrometric lateral resolution (~ 1.4 µm) took around 340 min (i.e., about 37 s for each energy). At the SXM end station, the single energy µ-XRF map recorded above the Ca K-edge from a region of interest of sample L_1_ (48 × 206 µm^2^) took approximately 10 min (Fig. 5a) and each full µ-XANES spectrum (549 energies) took about 1 min 40 s per point. They provide the most complete spectral information, but only on a limited number of scattered points.

**Table 1:** Experimental conditions used for performing calcium speciation investigations of the consolidated Lecce stone mock-up.

Method	Lateral resolution (h × v) (µm^2^)	Sample	Number of energies	Energy/energy range (eV)	Energy step size (eV)	Field of view/ map size (h × v) (µm^2^)	Pixel size (h × v) (µm^2^)	Pixel total number per energy scan	Acquisition time per energy scan (min:s)	Total acquisition time (min)
FF-XANES imaging (transmission mode)^a^	~ 1.4 × 1.4	L_1_, L_3_ (Figs. 3 and 4)	549	3,938–4,398	0.3–5	~ 1,000 × 1,000	0.65	~ 2.36‬ × 10^6^	~ 00:37	~ 340
μ-XRF mapping^b^	~ 0.7 × 0.3	L_1_ (Fig. 5a)	1	4,400	–	48 × 206	1.5 × 0.6	1.0987 × 10^4^	~ 10:00	~ 30
single point μ–XANES (XRF mode)^b^	~ 0.7 × 0.3	L_1_ (Fig. 5b)	549	3,938–4,398	0.3–5	–	–	–	–	~ 5^c^
FS μ–XANES mapping (XRF mode)^b^	~ 0.7 × 0.3	L_1_ (Figs. 5d,e and 6)	74	4,012–4,198	0.5–6	15 × 205	1 × 1	~ 3.075 × 10^3^	~ 2:00	~ 148
SIXES mapping	~ 0.7 × 0.3	L_1_ (Fig. 6)	8	4,051.9 4,053.1 4,056.1 4,056.9 4,057.2 4,064.0 4,066.1 4,198.0^d^	–	15 × 205	1 × 1	~ 3.075 × 10^3^	~ 2:00	~ 16

^a^Flux: ~ 10^12^ ph/s [unfocused beam, diameter (h × v): ~ 1.5 × 1.5 mm^2^].

^b^Flux: ≈ 2 × 10^10^ – 2 × 10^11^ ph/s [focused beam, diameter (h × v): ~ 0.7 × 0.3 µm^2^].

^c^Time required for acquiring 3 scans. Thus, for 1 scan the total acquisition time is of about 1 min 40 s.

^d^Each energy was selected for mapping the distribution of a specific Ca phase (in ascending order of energy): unknown, modified calcite, dolomite, ACC, Mg-rich CaCO_3_ phase, calcite, vaterite (see Supplementary Information, Figs. S2 and S4, for details). The map at 4,198.0 eV was used for normalization.

The content of information obtained by FF-XANES imaging was also achieved by performing the FS µ-XANES mapping investigation (Fig. 5d,e). However, in order to collect the dataset in a reasonable time and due to relatively high dwell times (40 ms per pixel; mainly limited by the electronics of the XRF detector), a stack of only 74 µ-XRF maps around the Ca K-edge was collected in a rather small region of interest of the sample (15 × 205 µm^2^; step size: 1 × 1 µm^2^). Despite the spectral resolution of the FS µ-XANES profiles (namely the number of energies of the image stack) is lower with respect to the spectra collected by single point XRF mode µ-XANES and FF-XANES imaging, a reliable discrimination among ACC, various CaCO_3_ polymorphs and other different Ca-based compounds, was achieved. Nevertheless, by keeping the same pixel size (1 × 1 µm^2^) and assuming to have analyzed an area of similar size (i.e., 1,000 × 1,000 µm^2^) with the same spectral resolution of the FF-XANES dataset (i.e., 549 images), the acquisition time for the FS µ-XANES mapping experiment would have been about 10^3^ times longer than FF-XANES imaging.

On the other hand, as a result of the worst lateral resolution, the FF-XANES datasets (lateral resolution of ~ 1.4 μm) show a lower definition than those recorded using the SXM setup (focused beam size down to 0.7 × 0.3 µm^2^). It follows that features with sizes of few micrometres (e.g., 5–10 µm in diameter) might be not clearly detected in the FF-XANES dataset.

Besides, FF-XANES has the strong constrain imposed by the transmission geometry, that limits the applicability of this technique to samples of appropriate composition (concentration of the element of interest) and appropriate sample preparation. Conversely, FS µ-XANES imposes far less constraints in terms of sample preparation and composition.

Thanks to the reduction of the number of energies (here by a factor of ~ 10), the SIXES mapping approach is much faster than FS µ-XANES, therefore allowing to cover larger regions of interest with submicrometric resolution. However, it should be noted that SIXES mapping has a fundamental drawback: it requires an *a-priori* knowledge of the sample composition, since the set of energies is determined based on a set of reference spectra. It can be a good primary approach to assess the heterogeneity of a sample and to determine the location of points of interest where single point µ-XANES spectra can then be acquired. SIXES mapping is also relevant to study several samples of similar compositions, but which vary by the distribution of these components^56^.

The differences observed between the FS µ-XANES and SIXES mapping (Fig. 6 and Supplementary Information, Fig. S4), both in terms of noise level and relative concentration of the various Ca-based compounds, are not only due to the limited number of energy points used in the SIXES approach, but also to the different employed normalization procedures. While for FF-XANES imaging and FS µ-XANES mapping, we used two linear functions in the pre- and the post-edge region to normalize the XANES stacks, for the SIXES mapping dataset the procedure was simplified to a normalization by a constant factor only in the post-edge region.

The Ca phase distributions obtained from the analysis of the same area of sample L_1_ using different approaches are rather similar for vaterite and calcite. Differences are instead visible in the shape, size and distribution of the Mg-rich grains localized inside the matrix. In particular, as Fig. 5 illustrates, the Mg-rich CaCO_3_ grain visible in the FS µ-XANES map is not visible in the corresponding FF-XANES image, where only calcite has been detected. This discrepancy is related to the different detection geometry of the FF-XANES (in transmission mode) and SXM (in fluorescence mode; incident angle: 62°, detection angle: 69°) end stations, to the heterogeneity of the sample throughout its thickness (that features the presence of Mg/Ca-crystals embedded in the calcite-based matrix) and to the different probed depth of the two techniques. In that respect, at the SXM end-station, the X-ray attenuation length at the Ca K-edge (4,038 eV) in the Lecce stone mock-up (assuming an average density = 2.7 g/cm^3^ and CaCO_3_ as main component) is estimated around 34 µm.

## Conclusions

In this work, we have successfully employed Ca K-edge 2D-XANES spectroscopy for solving the issue of determining at the submicrometer scale the nature and stratigraphic distribution of amorphous CaCO_3_ and various CaCO_3_ polymorphs (i.e., vaterite and calcite) associated to the application of a calcium acetoacetate-based consolidant in limestone substrates. Overall, the study provided accurate qualitative and semi-quantitative information on the newly formed calcium-based phases within the carbonatic matrix, with fully consistent results by using the following Ca K-edge 2D-XANES approaches : (i) the acquisition of speciation images in full-field transmission mode (FF-XANES), (ii) the standard acquisition of μ-XANES spectra in XRF mode at a limited number of locations combined with that of single energy μ-XRF maps, and (iii) the collection of speciation maps, in both hyper-spectral (FS µ-XANES) and multi-spectral (SIXES) mapping in XRF mode.

The pros and cons of such different 2D-XANES-based approaches have been evaluated by comparing analytical performances in optimized experimental configuration, so as to ultimately minimize risks for beam damage and efforts spent for sample preparation.

Notably, FF-XANES imaging in transmission mode has the great advantage of permitting to collect higher representative datasets with respect to the scanning µ-XRF mode, because of its capability of analyzing large areas (orders of mm^2^) and of measuring one XANES spectrum for each pixel of the multi-energy image stack in a feasible time span. On the other hand, the FF-XANES datasets are characterized by a lower lateral resolution, achieving values of the order of ~ 1–2 µm^2^. In the context of our research, the main limitation of the FF-XANES approach is due to the necessity of preparing cross-sections of suitable thickness as to work in transmission mode (which also implies that signal is averaged over the sample thickness). In this specific application, the brittle nature of the analyzed limestone substrate made very challenging the preparation of cross-sections thin enough (i.e., not thicker than 80 µm) for obtaining reliable results.

When it is impossible to manage the sample preparation as to obtain the suitable thickness for working in transmission, the XRF acquisition mode can be profitably exploited. For non-transparent Ca-samples, μ-XRF mapping in combination with single point μ-XANES analysis in XRF mode allows for obtaining meaningful Ca-speciation information at a higher lateral resolution [in this work: down to 0.7 µm (horizontal) and to 0.3 µm (vertical)]. The main drawback of such XANES-based approach is the low representativeness of the data, since the possibility of acquiring a small number of XANES spectra at few spots of the mapped area in a practicable time span.

FS μ-XANES mapping in XRF mode is a valid compromise between the transmission mode FF-XANES imaging and the single energy μ-XRF mapping coupled with XRF mode μ-XANES analysis at selected spots. With respect to the latter, it provides for more representative datasets (one XANES spectrum can be extracted from each pixel of the multi-energy image stack) with comparable high lateral resolution. With respect to transmission mode FF-XANES imaging, it enables to work on non-transparent samples. The main limitation remains still related to the long acquisition times which also determined the spectral resolution in term of number of energies of the image stack.

Finally, providing the sample composition is known *a-priori*, the SIXES mapping combines the advantages of the µ-XRF detection, together with the gain in speed. It is a very efficient way to highlight the main heterogeneities of the sample and to identify points or regions of interest where µ-XANES spectra or FS μ-XANES maps can then be acquired for an accurate determination of local concentrations of specific compounds. By reducing the number of energies compared to FS µ-XANES and single point µ-XANES, such approach may contribute to avoid/minimize possible risks of beam-induced damage events.

At beamline ID21, the increased flux offered by the new Extremely Brilliant Source (EBS) together with the refurbishment of XRF detectors and electronics should significantly increase the acquisition speed (target of 5–10 ms per pixel, in spite of 40–100 ms per pixel). This should definitively help in making SIXES and FS μ-XANES mapping in XRF mode standard techniques at high lateral resolution for speciation studies of non-transparent and heterogenous matrixes, such are those of different types of cultural heritage objects (e.g., consolidated stones, paintings, ceramics…) geological and biological samples.

## Supplementary information

Supplementary Information.
