# Supplementary material for: Synchrotron radiation Ca K-edge 2D-XANES spectroscopy for studying the stratigraphic distribution of calcium-based consolidants applied in limestones
Source: Sci Rep. 2020 Aug 31;10:14337. doi: 10.1038/s41598-020-71105-8 (PMC7459109; doi:10.1038/s41598-020-71105-8)
Supplement: Supplementary file 1 — Supplementary Information. [file 41598_2020_71105_MOESM1_ESM.docx]

**Supplementary Information**

**Synchrotron radiation Ca K-edge 2D-XANES spectroscopy for studying the stratigraphic distribution of calcium-based consolidants applied in limestones**

Letizia Monico^1,2,*^, Laura Cartechini^1,*^, Francesca Rosi^1^, Wout De Nolf^3^, Marine Cotte^3,4^, Riccardo Vivani^5^, Celeste Maurich^1^, Costanza Miliani^6^

^1^ Istituto di Scienze e Tecnologie Chimiche "Giulio Natta" (SCITEC), CNR, Via Elce di Sotto 8, 06123 Perugia, Italy.

^2^ SMAArt Centre and Department of Chemistry, Biology and Biotechnology, University of Perugia, Via Elce di Sotto 8, 06123 Perugia, Italy.

^3^ ESRF, Avenue des Martyrs 71, 38000 Grenoble, France.

^4^ L.A.M.S., CNRS UMR 8220, Sorbonne Université, UPMC Univ Paris 06, Place Jussieu 4, 75005 Paris, France.

^5^ Department of Pharmaceutical Sciences, University of Perugia, Via del Liceo 1, 06123 Perugia, Italy.

^6^ Istituto di Scienze del Patrimonio Culturale (ISPC), CNR, Via Cardinale Guglielmo Sanfelice 8, 80134 Napoli, Italy.

*** Corresponding Authors:** letizia.monico@cnr.it; laura.cartechini@cnr.it

**1. SR µ-XRD mapping**

SR µ-XRD mapping measurements of regions of interest of the not treated and of the consolidated Lecce stone mock-ups were performed at the scanning μ-XRD/μ-XRF end station hosted at beamline ID21 of the European Synchrotron Radiation Facility (ESRF, Grenoble, France). ^[^^[[1]](#endnote-1)]^

Investigations were carried out by means of a fixed exit double-crystal Si(111) monochromator with an incident beam of 8.5 keV, which was focused by means of a Kirkpatrick-Baez mirror system down to a size of ~2×2 μm² (h×v). μ-XRD patterns were recorded using a taper optics CCD FReLoN camera (2048×2048 pixels, pixel size 52 μm) and with an exposure time of 10 s per pixel.

Data processing has been done by XRDUA software. ^[^^[[2]](#endnote-2)]^

| 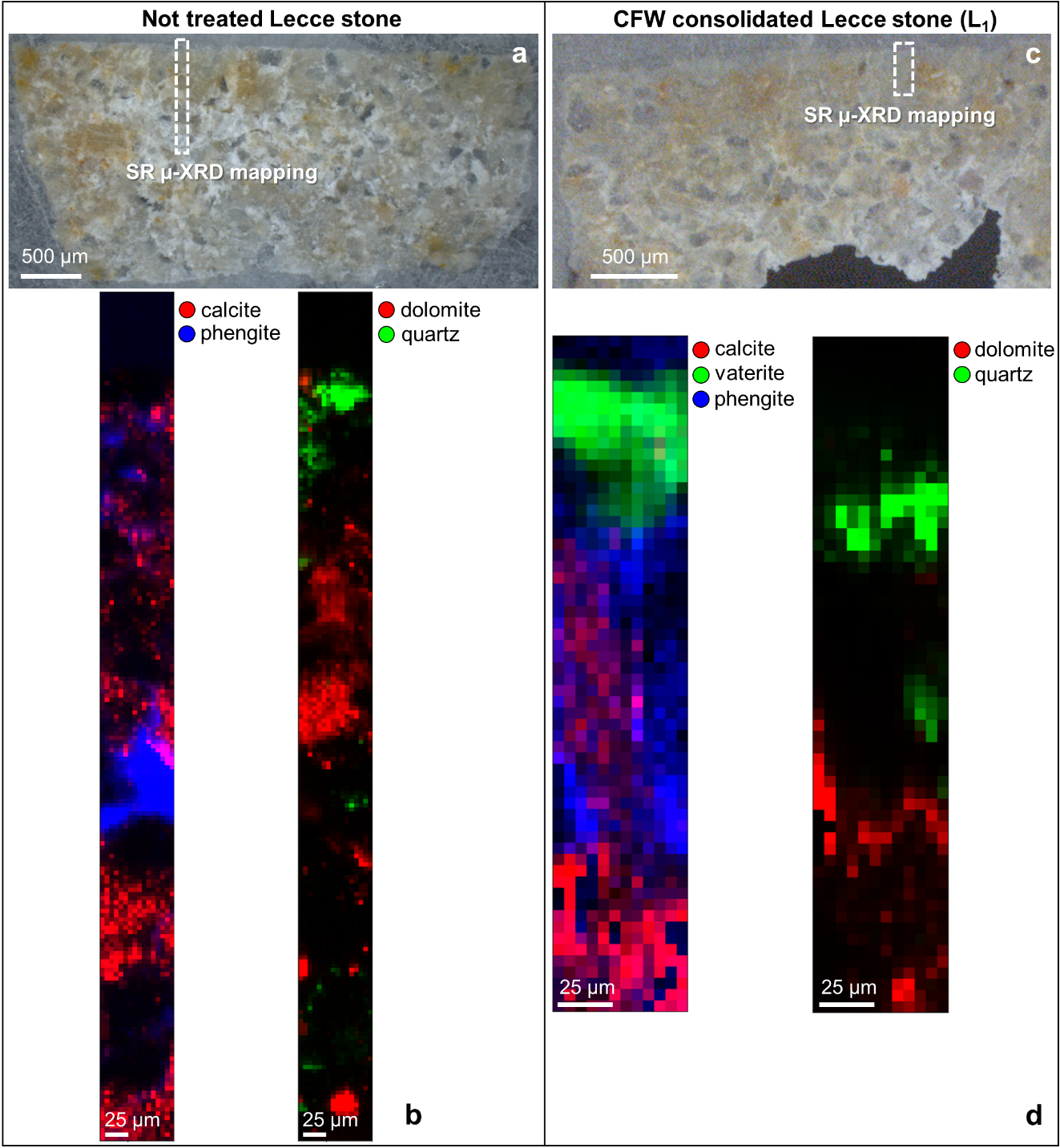 |
| --- |
| **Figure S1.** Microphotographs of the cross sections obtained from Lecce stone mock-ups **(a)** before consolidation and **(c)** after consolidation with CFW (RH=50%, T=40 °C, 5 months). **(b,d)** RGB SR µ-XRD distributions of (left) calcite/vaterite/phengite and (right) dolomite/quartz [energy: 8.53 keV; step size (h×v): 5×5 μm^2^; exposure time: 10 s per pixel]. Maps shown in **(b)** and **(d)** were recorded from the areas indicated by the white dotted rectangles reported in **(a)** and **(c)**, respectively (see text of main paper for details). Data were obtained by means of XRDUA. ^[2]^ |

**2. Ca K-edge XANES spectra of reference compounds**

Ca K-edge XANES spectra (both in transmission and XRF mode) of the reference compounds
(Fig. S2, black lines; see “Materials and methods” for details about the employed experimental conditions) were obtained from selected areas of the not treated Lecce stone mock-up (i.e., unknown, modified calcite, dolomite, Mg-rich CaCO_3_ phase) and from powders of two different Ca-based consolidant products stored under different RH conditions for 5 months, namely: i) a water-based consolidant formulation of calcium acetoacetate (denoted as CFW) and ii) the commercially available nanolime-based CaLoSil^®^ IP5 (IBZ-Salzchemie GmbH & Co.KG, Germany). Notably, the ACC spectrum was obtained from the analysis of the uncured calcium acetoacetate-consolidant powder, while that of vaterite from another portion of the same material aged at RH=80%. The spectral profile of calcite was instead recorded by investigating the Calosil^®^ IP5 powder treated at RH≥95%. Before performing the XANES analysis, bulk XRD and FT-IR measurements were carried out on the Ca-based consolidant powders (data not shown) for assessing their composition/purity.


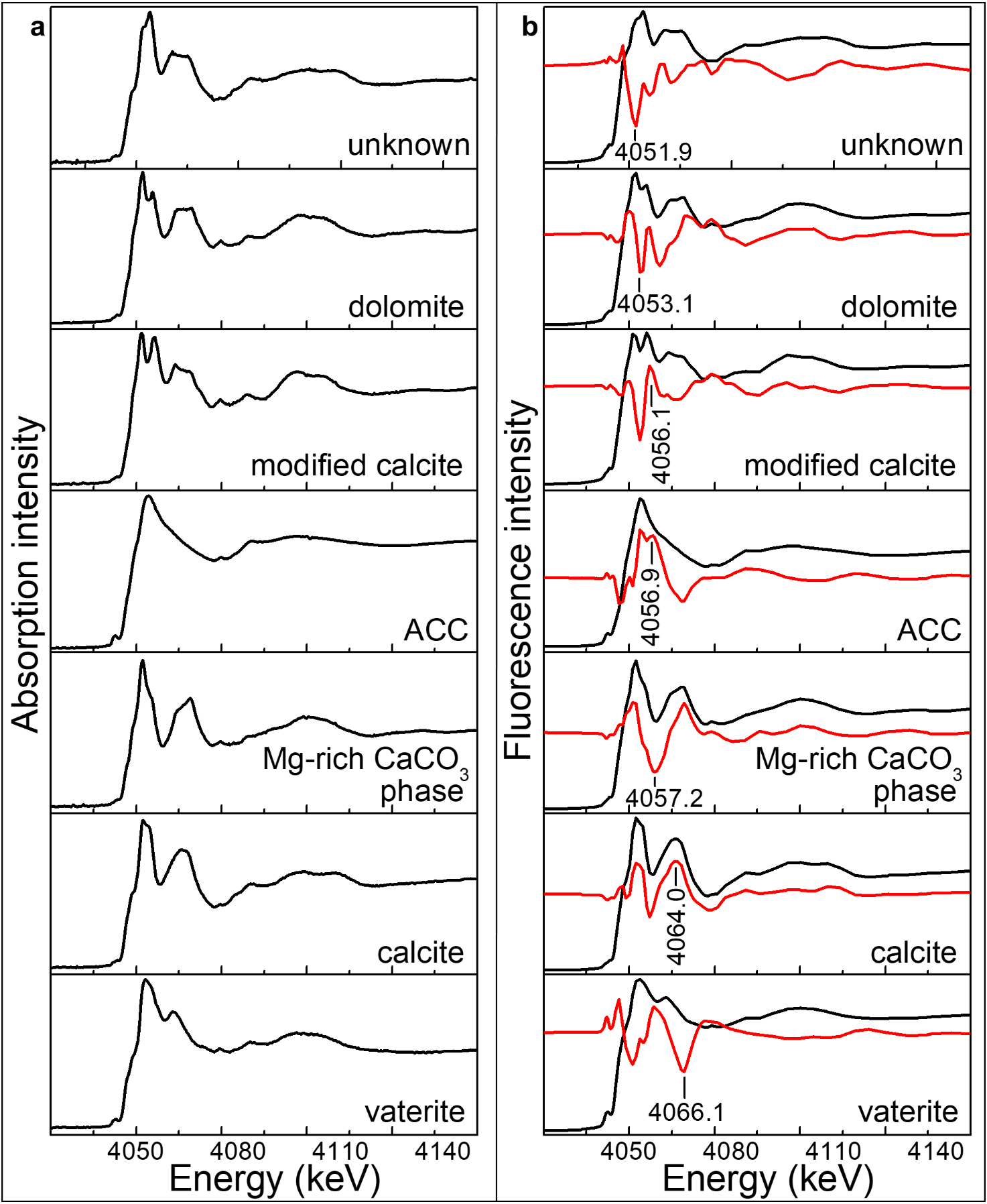


**Figure S2.** (black) Ca K-edge XANES spectra of the seven Ca-reference compounds recorded in **(a)** transmission mode and **(b)** XRF mode and used for the least squares linear combination (LSLC) fitting (*cf.* main paper, Figs. 2,3,5,6). In **(b)**, red lines represent the difference between each spectrum and the average of the other six spectra. The seven energies correspond to the first or second maxima (in absolute) and were selected for the SIXES mapping (*cf.* main paper, Fig. 6). XANES spectra were normalized using ATHENA. ^[^^[[3]](#endnote-3)]^

**3. Determination of the edge-jump, noise filtering and normalization**

In the first step of XANES imaging/mapping data-processing, pixels showing weak absorption (i.e., poor signal-to-noise ratios) were filtered using the absorption edge-jump, defined as the difference between the average intensity value in the post-edge region and the average intensity value in the pre-edge region. Pixels with edge-jumps smaller than a defined threshold value were removed (their intensity at all energies was set to zero; e.g., in the region where Si-grain are present – see Fig. S3a). The threshold was calculated for each pixel as the pre-edge standard deviation of that pixel (indicating the XANES noise level) multiplied by a user-defined factor. The obtained edge-jump maps (Fig. S3) show the distribution of Ca and provide information about the relative concentration differences within the analyzed field of view. In the FF-XANES imaging dataset, small local differences in the thickness of the sample may influence the result.

In a second step, an automated normalization procedure permitted to filter pixels for which the calculated pre- or post-edge lines reveal very high or very small slopes (less than 1% of all pixels have been removed). Further details about data processing are reported elsewhere. ^[3,^^[[4]](#endnote-4),^^[[5]](#endnote-5)]^

| 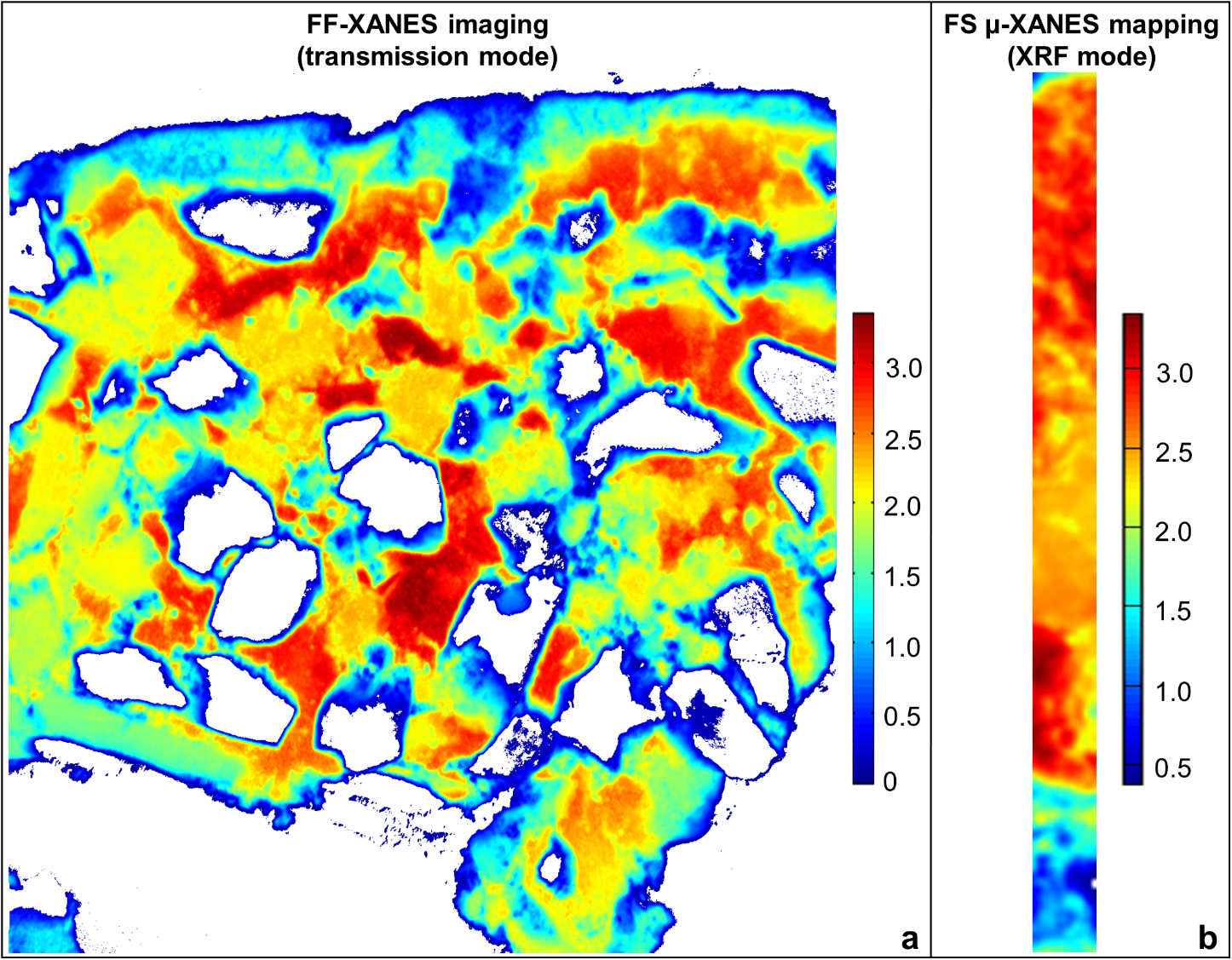 |
| --- |
| **Figure S3.** Ca K-edge-jump maps obtained from the processing of the **(a)** transmission mode FF-XANES imaging and **(b)** the XRF mode FS µ-XANES mapping datasets recorded from sample L_1_ (*cf.* main paper, Figs. 3 and 5d,e). Data were processed by means of TXM-Wizard. ^[4]^ |

**4. Ca K-edge XANES analysis of sample L_1_: least squares linear combination (LSLC) fit results**

To obtain semi-quantitative information about the relative abundances of the Ca phases present in the sample, least squares linear combination (LSLC) fitting of each single-pixel/single-point XANES spectrum was performed using the set of Ca-reference profiles shown in Fig. S2. Based on what described in previous studies, ^[3-5]^ the quality of each LSLC fit was checked taking into account the R-factor, chi-square and reduced chi-square values.

In Table S1 an overview of the LSLC fit results for a selection of the Ca K-edge XANES spectra reported in the main paper are reported (*cf.* Figs 3 and 5).

| **Table S1**. LSLC fit results of a selection of the Ca K-edge XANES spectra obtained from calcite-, vaterite- and ACC-based areas of sample L_1_ by means of different 2D XANES-based approaches. | | | | | | | | | | | |
| --- | --- | --- | --- | --- | --- | --- | --- | --- | --- | --- | --- |
| **XANES spectrum** | **Figure n.** | **Component weight (%)** | | | | | | | **Fit error** | | |
|  |  | vaterite | calcite | ACC | dolomite | modified calcite | unknown | Mg-rich CaCO_3_ phase | Chi-square | R-factor | Reduced chi-square |
| **FF-XANES imaging** |  | | | | | | | | | | |
| pt. 01 | Fig. 3e | 56±5 | 37±5 | 7±3 | - | - | - | - | 0.349 | 0.00090 | 0.00083 |
| pt. 02 |  | 75±8 | 15±5 | 10±5 | - | - | - | - | 0.223 | 0.00056 | 0.00053 |
| **Single point**  **µ-XANES** | | | | | | | | | | | |
| pt. 01 | Fig. 5a | 70±5 | 15±5 | 15±5 | - | - | - | - | 0.066 | 0.00021 | 0.00017 |
| **FS µ-XANES**  **mapping** | | | | | | | | | | | |
| pt. 01 | Fig. 5e | 75±5 | - | 15±5 | - | - | 10±5 | - | 0.052 | 0.00086 | 0.00076 |
| pt. 03 |  | - | 80±5 | 20±5 | - | - | - | - | 0.107 | 0.0015 | 0.0016 |
| pt. 07 |  | 9±5 | 36±5 | 46±5 | - | - | 9±5 | - | 0.100 | 0.0015 | 0.0015 |

**Determination of SIXES energies and comparison between the concentration maps obtained by FS µ-XANES and SIXES mapping**

The SIXES approach consists in reducing the spectral axis into a list of few energies. ^[^^[[6]](#endnote-6)]^ Here, it was decided to reduce this list to the minimum (i.e., seven energies plus one for normalization). To determine these energies, the Ca K-edge XANES spectrum of each reference compound was compared to the average spectral profiles of the other references (Fig. S2, red lines). For each reference, we determined the energy by maximizing the absolute difference. The final list of seven energies (Fig. S2 and main paper, Table 1) was established by considering the energy value giving the maximum contrast for each reference and, in case of redundancy, the second maximum (in our case: ACC, dolomite and modified calcite, which have their maximum at the same energy).

To assess the quality of the LSLC fit results of the SIXES mapping, for each pixel of the seven Ca-phase maps we calculated the difference (in absolute) of the concentration related to the FS µ-XANES and SIXES datasets (*cf.* main paper, Fig. 6). Fig. S4 presents the results in the form of cumulated population (in percent), for different ranges of differences. The graph shows that for all Ca-based species, except vaterite and the unknown phase, more than 85% of the pixels give a difference lower than 0.2. The quantification of vaterite and the unknown species is less reliable in those regions where the relative abundance of vaterite is higher.


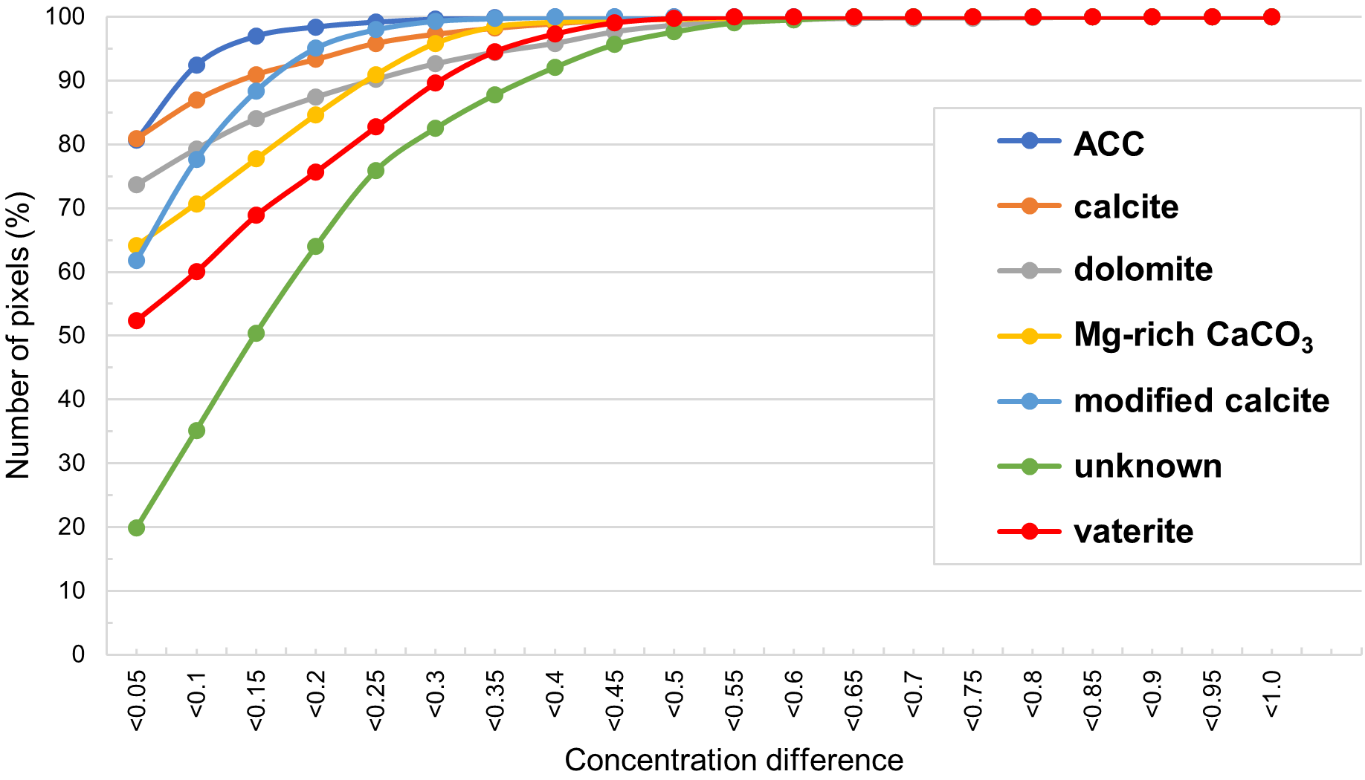


**Figure S4.** Difference of concentrations of Ca-based species as determined by LSLC fit of the FS µ-XANES and SIXES mapping datasets. For each compound, results are presented in percentage of the number of pixels over the map.

**References**

1. [] Cotte, M. *et al.* The ID21 X-ray and infrared microscopy beamline at the ESRF: status and recent applications to artistic materials. *Journal of Analytical Atomic Spectrometry* **32**, 477-493 (2017). [↑](#endnote-ref-1)
2. [] De Nolf, W., Vanmeert, F. & Janssens, K. XRDUA: crystalline phase distribution maps by two-dimensional scanning and tomographic (micro) X-ray powder diffraction. *Journal of applied crystallography* **47**, 1107-1117 (2014). [↑](#endnote-ref-2)
3. [] Ravel, B. & Newville, M. ATHENA, ARTEMIS, HEPHAESTUS: data analysis for X-ray absorption spectroscopy using IFEFFIT. *Journal of synchrotron radiation* **12**, 537-541 (2005). [↑](#endnote-ref-3)
4. [] Liu, Y. *et al.* TXM-Wizard: a program for advanced data collection and evaluation in full-field transmission X-ray microscopy. *Journal of synchrotron radiation* **19**, 281-287 (2012). [↑](#endnote-ref-4)
5. [] Meirer, F. *et al.* Full-field XANES analysis of Roman ceramics to estimate firing conditions-A novel probe to study hierarchical heterogeneous materials. *Journal of Analytical Atomic Spectrometry* **28**, 1870-1883 (2013). [↑](#endnote-ref-5)
6. [] Sakurai, K., Iida, A., Takahashi, M. & Gohshi, Y. Chemical State Mapping by X-Ray Fluorescence Using Absorption Edge Shifts. *Japanese Journal of Applied Physics* **27**, L1768-L1771 (1988). [↑](#endnote-ref-6)
